# Malformation of the Brain

**Published:** 1848-04-01

**Authors:** A. Wigan


### CASE OF MALFORMATION OF THE BRAIN, WITH INFERENCES.

COMMUNICATED BY THE LATE A. WIGAN, ESQ., M.D.

[The history of the following case was drawn up by Dr. Wigan, a few days previously to his death, during a temporary sojourn at Sussex House, Hammersmith, the residence of the Editor. More than ordinary interest will attach to the paper, when it is known that it was the last professional article which this distinguished medical psychologist penned.—Editor.]

The following case, on which I propose to found a few suggestive observations, is not selected on account of its rarity, nor yet that it is the most extreme form of abnormal structure. I have chosen it from a great number of similar examples of deformity of the cranium, which have passed before my eyes, simply because it admits of being readily authenticated. No man of a philosophical turn of mind will set himself to reason on a subject till he has entire confidence in the fidelity with which it is stated. He is more especially sceptical if the narrator have a new theory to advocate, because he knows that the most veracious may be insensibly biassed, and unconsciously convey an erroneous impression of the facts.

About three years ago, I was asked by a medical practitioner in Surrey, to examine the head of his servant-boy, then about fifteen years of age, whom he had taken out of the poor-house, from pure compassion—the lad being of weak intellect, and (as a matter of course) an object of ridicule and ill-treatment by his companions, in consequence of his inability to perform any of the ordinary mental exercises, if in the slightest degree hurried. On these occasions, he became speedily unconscious and utterly lost.

A medical gentleman, one of the most kind-hearted of human beings, determined to rescue this poor boy from the fate which awaited him, and to try if gentle management, patience, and good food, might not gradually bring out such a development of intellect as would enable him to earn his living. He was more especially excited to this, by observing that the boy's moral qualities and natural impulses were good, and that he was able to accomplish many of the common intellectual processes, if time were given him slowly to comprehend, and then time to arrange and prepare his answer. A lady of middle age, residing in the house, a relation of the family, volunteered to give him the rudiments of education befitting his station in life.

Gradually and slowly he learnt to read tolerably, and to write legibly, but he was for some time unable to comprehend the simplest arithmetical process; to follow even the explanation of it, or to count so far as ten. The slightest hasty word, or attempt to quicken his tardy mental efforts, produced instantaneous confusion, and entire inability to think, or to guide his actions—and it was some time before his agitation could subside.

When I first saw him, the external appearance of the head was extraordinary and curious—the great inequality between the two halves of the cranium was manifest to the most careless observer, in spite of a profusion of hair. It had, indeed, been noticed from the beginning, but was, I believe, simply regarded as "deformity," till my speculations attracted some attention.

On examining the head, it gave exactly the impression, as if the left cerebrum had been cut off by the blow of a sabre, in a line from the coronal suture to the ear, and then covered with a flat bone to complete the cranium. I am certainly within the bounds of truth, in saying, that the inequality was fully two-thirds—indeed so closely did that half of the skull approach the corpus callosum, that there seemed scarcely any space left for the convolutions on that side. The defective hemisphere could not have been more than one-third the size of the other, which was large and well formed. The boy walked in rather a shambling manner, but not more so than we often see in persons of his class, and certainly there was nothing which could be denominated paralysis. The body was well formed and vigorous for his age, the countenance regular and physically handsome, but the look was vacant and heavy, and there was an occasional slight want of parallelism in the eyes. He was indolent, but that was, I think, chiefly from the excessive indulgence of his master and mistress, and he was disinclined to talk. He was still, however, an object of banter and ridicule with his fellow-servants—but on explaining to them the nature and cause of his unhappy peculiarities, they very properly ceased to make him the butt of their ridicule.

After a long interval, caused principally by my absence from England, I saw the youth again a short time ago. He is now in his eighteenth year, and has continued steadily to improve in physical growth and health, with a *pari passu* increase of intellectual power and self-possession. He is as active as required, though not so active as he ought to be, and is in person a fine, powerful, good-looking, full-grown man.

Along with this improvement in his intellect, a corresponding growth of brain has taken place in that hemisphere whose progress had been so long arrested. The deformity of the skull seems, on a superficial examination, almost obliterated, so that unless the profusion of hair were removed, it would require to handle the skull to perceive it. Any one acquainted with the anatomy of the part would, however, easily be convinced that one brain is still nearly a third less than the other.

There is every reason to believe that this young man will attain the ordinary average of intellect; but it is probable that the head will always retain some deformity, although not perhaps greater than we see in many of the insane who had not in early life shown any signs of defective intellect.

When the boy was first taken from the workhouse, he could just accomplish words of one syllable, but could not even approach the comprehension of arithmetic. The slightest hurry or agitation threw him into a state of utter confusion and mental annihilation.

As his education slowly advanced, it was remarked that on some days he could master easily lessons, which the next day he could not in the slightest degree comprehend, although he had a strong wish to learn. The lady very prudently on such occasions always dismissed him at once, and did not permit him to continue his abortive attempts. My hypothesis on this subject is, that he had always one brain in a state for mental exercises, but that, in the gradual advance of the other, it produced confusion by the occasional admixture of its imperfect and incomplete mentation with the comparatively correct intellectualism of its fellow. The two could not be in unison, and therefore could not give the *one mind* of a perfect reasoning apparatus.

It is not surprising that the great difference in the weight of the two hemispheres should produce an inclination of the head to one side. It is, and always has been, carried with leaning to the right, the larger brain.

The present state of this young man is that of perfect health and vigour; but he is slow in all his movements. He rarely speaks more than a few words, and never but when there is positive need of it. He writes and reads well, and has mastered thoroughly the first four rules of arithmetic. He has voluntarily made himself acquainted with the names of the drugs in their abbreviated form—sets himself to write new labels with great neatness—is a very accurate, but very slow dispenser in all the things entrusted to him, and volunteers to write labels of instruction on the phials, though such duties are neither required nor expected of him. His disposition is rather sullen—easily offended at anything said by his fellow-servants, and brooding over it for two or three days, refusing his food, &c. I am, however, much inclined to attribute this aptitude to take offence to the excessive indulgence with which he has been treated, and to *overfeeding*. Altogether, he is now considerably in advance of the average of youths of his own age and station.

I firmly believe that if this boy had been left in the workhouse, or had been placed under a tradesman (who would have treated him as obstinate and stupid, and used threats and coercion), he would long ago have become a confirmed idiot.

A great number of cases, which are forced by ill treatment, or allowed to degenerate into idiocy, are, I believe, caused by this inequality in the development of the two brains. I encountered at Lucerne, last year, Dr. Stahl, grandson of the celebrated *phlogiston* Stahl, and brother of the eminent Prussian jurist. He had with him a collection of skulls of Cretins, showing the great inequality in size and form of the two halves of the cranium, and consequently of the two cerebra. On this subject, he had written a paper in a leading German journal, which had attracted a great deal of notice. Unfortunately, I could not speak German, and he knew no language but his own, so that our intercourse would have been very limited, but for the intervention of the celebrated M. Valentin, the physiologist, who is professor at Berne, and who invited a large party to his house to hear me explain my ideas on the subject of the duality of the mind—that is, the completely independent action of the two hemispheres.

I am far from asserting that inequality in the size and form of the hemispheres is the main or most frequent cause of idiocy, or even of imperfect mind; nevertheless, it is so common, that it should always be borne in mind, and the fact thoroughly ascertained, for a much less inequality than is here narrated may retard or prevent the due development of the understanding; and if defective intellect depend on this cause, it is a great thing to have an entire conviction that gentle treatment and time can alone accomplish the cure. In every case of defective or retarded intellect, then, let the head be attentively examined—and this will be done more completely, and with a more certain result, by shutting the eyes, and trusting only to the taxis. The countenance deceives sometimes by its expression and the arrangement of the hair, but any man who possesses (as the phrase is,) "eyes at his finger ends," can form to himself a true idea of shape and relative magnitude. He who cannot is unfit for the task.

